# Noah’s Ark Conservation Will Not Preserve Threatened Ecological Communities under Climate Change

**DOI:** 10.1371/journal.pone.0124014

**Published:** 2015-04-16

**Authors:** Rebecca Mary Bernadette Harris, Oberon Carter, Louise Gilfedder, Luciana Laura Porfirio, Greg Lee, Nathaniel Lee Bindoff

**Affiliations:** 1 Antarctic Climate and Ecosystems Cooperative Research Centre (ACE CRC), University of Tasmania, Hobart, Australia; 2 Resource Management and Conservation Division, Department of Primary Industries, Parks, Water & Environment, Hobart, Australia; 3 Fenner School of Environment and Society, Australian National University, Canberra, Australia; 4 Institute for Marine and Antarctic Studies (IMAS), University of Tasmania, Hobart, Australia; 5 ARC Centre of Excellence for Climate Systems Science, University of New South Wales, Sydney, Australia; University of Massachusetts, UNITED STATES

## Abstract

**Background:**

Effective conservation of threatened ecological communities requires knowledge of where climatically suitable habitat is likely to persist into the future. We use the critically endangered Lowland Grassland community of Tasmania, Australia as a case study to identify options for management in cases where future climatic conditions become unsuitable for the current threatened community.

**Methods:**

We model current and future climatic suitability for the Lowland Themeda and the Lowland Poa Grassland communities, which make up the listed ecological community. We also model climatic suitability for the structurally dominant grass species of these communities, and for closely related grassland and woodland communities. We use a dynamically downscaled regional climate model derived from six CMIP3 global climate models, under the A2 SRES emissions scenario.

**Results:**

All model projections showed a large reduction in climatically suitable area by mid-century. Outcomes are slightly better if closely related grassy communities are considered, but the extent of suitable area is still substantially reduced. Only small areas within the current distribution are projected to remain climatically suitable by the end of the century, and very little of that area is currently in good condition.

**Conclusions:**

As the climate becomes less suitable, a gradual change in the species composition, structure and habitat quality of the grassland communities is likely. Conservation management will need to focus on maintaining diversity, structure and function, rather than attempting to preserve current species composition. Options for achieving this include managing related grassland types to maintain grassland species at the landscape-scale, and maximising the resilience of grasslands by reducing further fragmentation, weed invasion and stress from other land uses, while accepting that change is inevitable. Attempting to maintain the status quo by conserving the current structure and composition of Lowland Grassland communities is unlikely to be a viable management option in the long term.

## Introduction

Distributions of species and communities are shifting due to changing climatic conditions [1–4], and attempts to maintain the status quo in terms of species composition may be futile in many regions of the world. Variability through time is an inherent part of ecosystem behaviour, but conservation management frequently relies on a static view of ecosystems [5]. Listed ecological communities are bound by environmental legislation that sets thresholds for acceptable condition, species richness and composition. For conservation management to be effective in the future, it will need to accommodate changing ecological dynamics. Options that enable the diversity, structure and function of ecosystems to be maintained will need to be identified, rather than attempting to preserve current species composition.

Temperate grasslands are among the most threatened vegetation types on Earth, since they occur in those environments most amenable to human settlement and agriculture [6]. In Australia, lowland temperate grasslands are a national conservation priority, with less than 1% of their original extent remaining [7]. Lowland temperate grasslands are dominated by native grasses, with few or no emergent woody species. The dominant grasses are tussock-forming, and a variety of small forbs or wildflowers and non-tussock forming grasses and sedges occur in the inter-tussock spaces. Remnant patches in good condition are species-rich and are important habitat to a diverse array of flora and fauna, many of which are also listed as vulnerable or threatened.

Tasmanian Lowland Temperate Native Grasslands (LNGT) are listed as Critically Endangered on the *Environment Protection and Biodiversity Conservation Act 1999*, the Australian Government’s national environmental legislation. Less than 10% of the natural extent of this community remains [8] and most is on privately-owned land. The LNGT are comprised of two main floristic types, determined by the dominant grass species. The Lowland Themeda Grassland community (GTL) is dominated by *Themeda triandra* (Kangaroo grass) and the Lowland Poa Grassland community (GPL) is dominated by *Poa labillardierei* (Tussockgrass). *Austrodanthonia* spp. (Wallabygrass) and/or *Austrostipa* spp. (Speargrass) may also be present, along with a diverse assemblage of herbs and forbs, and few woody or shrubby species.

Native grassland communities in Tasmania are often represented by small, highly fragmented remnants, which vary greatly in condition due to past land uses such as grazing, burning and use of fertilisers. Current condition is likely to be important for the long-term viability of a community, with grasslands in better condition more resilient to change in the short term, and more adaptable in the long term, due to their greater genetic, floristic and structural diversity [9–12].

Management of threatened ecological communities requires some knowledge of where climatically suitable habitat is likely to persist into the future [13]. In contrast to the common approach of modelling changes to the distribution of a single species of interest under climate change, we model the distribution of a listed ecological community as a whole. Where dominant species are widespread, but the community has a highly restricted distribution, modelling climatic suitability for the dominant species will tend to overestimate the extent of suitable climate for the community. Modelling the extent of the community is more likely to capture important limiting factors and interactions [14].

Average temperatures in Tasmania are projected to increase by 2.6 to 3.3°C by the end of this century under a high emissions scenario (A2) [15, 16]. Temperature change is projected to be fairly uniform across Tasmania, and broadly similar across seasons. In contrast, total annual rainfall is not projected to change, but there are significant changes in the spatial pattern and seasonality of rainfall. Annual rainfall is projected to increase over coastal regions, and decrease in parts of northwest and central Tasmania where the grasslands currently occur. In these areas, autumn rainfall is projected to increase significantly after 2050, and decrease in spring. The suitability of future climatic conditions for temperate grasslands may therefore be influenced by changes to seasonality and interactions between climate variables, in addition to changes to mean conditions. Shifts in the floristics, structure and phenology of grassland communities, with a consequent loss of habitat and changed composition of species assemblages, are expected to occur as a direct response to the changing climate [10]. Other, indirect effects are likely, including changes in soil properties and nutrient cycling, changes to fire regimes, increased risk of weed invasion, and changes in land use and management, including “drought-proofing” through irrigation and enterprise diversification such as new crops and carbon plantings.

There are several vegetation communities similar in composition and geographic extent that are likely to be important in the long-term conservation of the LNGT. Firstly, the Lowland Grassland Complex (GCL) includes grasslands that are not classified as LNGT because they have insufficient cover of *Poa labillardierei* or *Themeda triandra*. They have a significantly greater extent than LNGT, and may play an important functional role in maintaining grassland species across the landscape. GCL are mostly derived from prior clearing of native grassy, dry woodland and forest communities or from eucalypt dieback events [17]. Secondly, the *Bursaria-Acacia* woodland and scrub community (NBA) grades with LNGT but is excluded from LNGT where *B*. *spinosa* exceeds 30% cover. This community has an herb-rich grassy understorey dominated by *Themeda triandra* with emergent *Bursaria spinosa* shrubs (up to 3–4 m). The structure of NBA is often an artefact of fire and grazing history, and increased frequency of burning can promote grassland vegetation and reduce shrub cover. Grasslands form a continuum with grassy woodlands dominated by various *Eucalyptus* species, which could be an important component of the management matrix if managed to maintain or favour grasslands.

In this paper we identify possible options for managing threatened communities under climate change, using the Lowland Grassland community (LNGT) in Tasmania, Australia as a case study. Firstly, we test whether future climate conditions are likely to remain suitable for the lowland grassland communities which make up the LNGT (GTL and GPL), and for their structurally dominant species. We also model current and future climatic suitability for closely related grassland (GCL) and woodland communities (NBA) in order to identify options for management in the event that future climatic conditions become unsuitable for the current lowland grassland community.

## Methods

### Ethics Statement

No ethics approval or permissions to access private or public lands were required for this study.

### Community and Species Distribution Modelling

Climatic suitability was modelled for the two grassland communities that make up the LNGT, the Lowland Themeda Grassland community (GTL) and the Lowland Poa Grassland community (GPL), as well as related grassy communities, the *Bursaria-Acacia* woodland and scrub community (NBA) and the Lowland Grassland Complex (GCL). An understanding of the distribution of each community, in addition to its structurally dominant species, is necessary for the practical management and conservation of a listed community under changing conditions.

GTL and GPL were modelled separately because they are expected to respond differently to changing climate variables due to the different photosynthetic pathways employed by their structurally dominant species. *Poa labillardierei* is a C3 grass, while *Themeda triandra* is a C4 grass. In general, it is expected that global warming will increase the dominance of C4 grasses, which have higher water efficiency, greater drought tolerance, and greater persistence in warmer climates than C3 species [18]. However, the effect of elevated CO_2_ and competitive interactions between C4 and C3 plants under different nutrient conditions and seasonality are not well understood [19, 20].

Presence locations for each of the grassland communities were generated by randomly sampling 1000 points from within the existing distribution of each of the community types from the state-wide digital vegetation map, TASVEG3.0 [21]. Although grasslands covered a larger extent prior to European settlement in the early 19^th^ century, and preferential clearing may have occurred in the past on more fertile soils or areas with fewer trees, the remaining grasslands are distributed within the likely extent of the original, so sampling the current distribution is a reasonable approximation to the range of suitable climatic conditions.

The climatic suitability for the two structurally dominant grasses, *Poa labillardierei* and *Themeda triandra*, were modelled under current and future climate conditions to assess the extent to which the composition of the grassland communities might change under future climate conditions. Locality data for the Australian geographic extent of these species were downloaded from the online database Atlas of Living Australia (http://collections.ala.org.au/public). After excluding spatially suspect records, there were 19,313 unique observations from the years 1770 to 2014 for *Themeda triandra*, and 5,755 observations for *Poa labillardierei* var. *labillardierei* from the period 1853 to 2013.

The Maxent model [22] was used to project the distribution of climate suitable for the grassland communities and species under current and future climate conditions. Maxent is a Species Distribution Model (SDM), or climate envelope model, which uses presence only data. SDMs are based on the statistical relationship between environmental or climatic variables and the current, observed distribution of a species or community. Assuming that this relationship remains unchanged, the future distribution of the species is then projected according to future climatic conditions. Other modelling approaches are available, including Boosted Regression Trees, Generalized Linear Models and Generalized Additive Models, and ensembles of these are sometimes used to represent the range in SDM outputs [23–25]. We chose Maxent because it is has been shown to perform well in comparison to several other models when there are few presence records available [26]. For each model run, ten replicate Maxent runs were calculated by cross-validation and using default values for all other parameters (e.g. regularisation). Relative occurrence probabilities above 0.5 were considered suitable, following Phillips & Dudík [27].

Choice of explanatory variables has an important influence on the outcome of species distribution models [28–31]. We use models based on climate variables to project changes to the communities and species’ potential climate domain. Our results therefore indicate relative climatic suitability rather than habitat suitability. In the absence of knowledge about the specific variables which determine grassland distributions in Tasmania, we used saturated models based on the full set of thirty five commonly used bioclimatic variables and allowed Maxent to perform variable selection. Maxent has been shown to be robust to correlated variables [32], and avoids over-fitting by applying a regularization penalty for each term included in the model [22, 33, 34]. We used the default regularization settings, based on values determined as optimal in empirical tuning [27].

### Future Climate Projections

Future climate conditions were taken from a dynamically downscaled regional climate model, the Conformal Cubic Atmospheric Model (CCAM), developed by the Commonwealth Scientific and Industrial Research Organisation (CSIRO), Australia. Six CMIP3 global climate models (ECHAM5/MPI-OM, GFDL-CM2.0, GFDL-CM2.1, UKMO-HadCM3, CSIRO Mk3.5 and MIROC3.2 (medres)) were downscaled to ~10km resolution by the Climate Futures for Tasmania project. These models were chosen because they represent current south-east Australian climate means and variability well [35], and cover the spread of projected rainfall change in southeast Australia present in the CMIP3 set of models [36]. Details of the modelling can be found in Corney *et al*. [37, 38], and the modelled projections are available through the Tasmanian Partnership for Advanced Computing (TPAC) portal (https://dl.tpac.org.au/tpacportal/).

We present results based on the A2 emissions scenario because global emissions are currently tracking at the higher level of this scenario [39]. The A2 scenario is broadly similar to the Representative Concentration Pathway (RCP) 8.5, which replaced the Special Report on Emissions Scenarios (SRES) emissions scenarios in the Intergovernmental Panel on Climate Change’s (IPCC’s) fifth assessment report (AR5) and Phase 5 of the Coupled Model Inter-comparison Project (CMIP5). Regional comparisons of projections from the CMIP5 and CMIP3 models have found surface temperature, wind and rainfall patterns to be highly consistent between the archives [40–42]. The release of the CMIP5 archive models has therefore not made the CMIP3 models redundant.

Current baseline climate surfaces were obtained from ANUCLIM version 6.1 [43], based on the 0.01° (~1 km) Digital Elevation Model for Australia. Climate change grids for maximum and minimum temperature (Tmax, Tmin), precipitation and pan evaporation were calculated relative to the ANUCLIM baseline (1976–2005; 1970–1995 for evaporation), for two future periods representing 2040–2069 and 2070–2099 (hereafter 2050 and 2080, the mean values of the 30 year periods). ANUCLIM was used to interpolate the regional climate model output to 1km and generate monthly mean data and the thirty-five bioclimatic variables for the current and future periods (Table 1).

**Table 1:** The set of bioclimatic variables used in the Maxent models of grassland communities and species.

Bio ID	Bioclimatic parameters
bio1	Annual Mean Temperature
bio2	Mean Diurnal Range (Mean(period max-min))
bio3*	Isothermality (bio2/bio7)
bio4	Temperature Seasonality (Coefficient of Variation)
bio5	Max Temperature of Warmest Period
bio6	Min Temperature of Coldest Period
bio7	Temperature Annual Range (bio5-bio6)
bio8	Mean Temperature of Wettest Quarter
bio9	Mean Temperature of Driest Quarter
bio10	Mean Temperature of Warmest Quarter
bio11	Mean Temperature of Coldest Quarter
bio12	Annual Precipitation
bio13	Precipitation of Wettest Period
bio14	Precipitation of Driest Period
bio15	Precipitation Seasonality (Coefficient of Variation)
bio16	Precipitation of Wettest Quarter
bio17	Precipitation of Driest Quarter
bio18	Precipitation of Warmest Quarter
bio19	Precipitation of Coldest Quarter
bio20	Annual Mean Radiation
bio21	Highest Period Radiation
bio22	Lowest Period Radiation
bio23	Radiation Seasonality (Coefficient of Variation)
bio24	Radiation of Wettest Quarter
bio25	Radiation of Driest Quarter
bio26	Radiation of Warmest Quarter
bio27	Radiation of Coldest Quarter
bio28	Annual Mean Moisture Index
bio29	Highest Period Moisture Index
bio30	Lowest Period Moisture Index
bio31*	Moisture Index Seasonality (CV)
bio32	Mean Moisture Index of Highest Quarter MI
bio33	Mean Moisture Index of Lowest Quarter MI
bio34	Mean Moisture Index of Warmest Quarter
bio35	Mean Moisture Index of Coldest Quarter

* bio3 (isothermality) can be interpreted as the evenness of temperature over the course of a year, or a quantification of how large the day-to-night temperature oscillation is in comparison to the summer-to-winter oscillation. A value of 100 would represent a site where the diurnal temperature range is equal to the annual temperature range.

**bio31 was not used because the Coefficient of Variation could not be calculated in areas where the standard deviation was zero (large areas of western Tasmania).

### Grassland Condition

Listing of the grassland community under the *EPBC Act 1999* requires an assessment of grassland condition to identify the remnant patches of greatest conservation value. For this analysis, grasslands that met at least six of the seven criteria for listing of LNGT under the *Environment Protection and Biodiversity Conservation Act 1999* were considered to be in good condition. These grasslands have been verified via field validation and/or aerial photo interpretation as meeting all of the criteria for listing (i.e. sufficient patch size, grass tussock cover, species type and woody plant cover) except for one criterion relating to minimum wildflower species richness. This criterion was not considered essential to qualify as being in good condition, because species richness in Tasmanian grasslands is often underestimated unless field validation is carried out in the peak flowering time. Table 2 shows the extent of each community considered to be in good condition.

**Table 2:** Extent of current GTL and GPL grasslands in good condition.

	Area of current extent (ha)	Area of good condition (ha)	% of current area in good condition
Lowland Themeda grasslands (GTL)	7,535	6516	87
Lowland Poa grasslands (GPL)	13,617	7920	58
Total lowland grasslands (GPL+GTL)	21,152	14,436	68

## Results

The results are presented as maps of relative climate suitability for the current period (in grey), and the two future periods (2050 in pink and 2080 in red). Future climate suitability is shown as the sum of all areas projected to be suitable by any climate model, the bounding box (Araújo and New 2007, Porfirio et al. 2014). This is likely to be a conservative estimate of future declines in suitable climate, as individual models projected substantially smaller areas in all cases. Black indicates the areas where all climate models agree that currently suitable climate will persist by 2080 (“persisting climate”).

### Suitability of future climate for the grassland communities

#### Lowland Themeda Grassland community (GTL)

**Fig 1:**
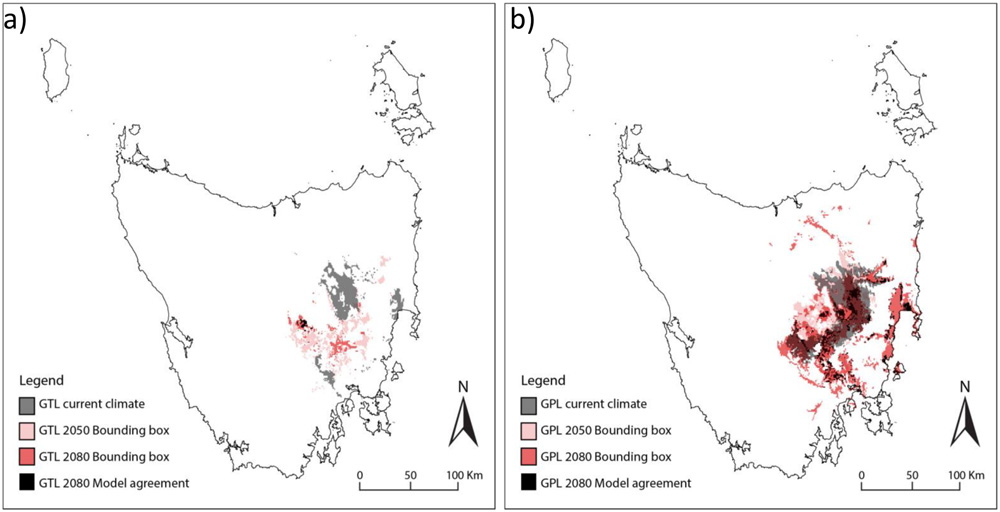
Current and future climate suitability for a) Lowland Themeda Grassland (GTL) and b) Lowland Poa Grassland (GPL) communities. White represents areas that are not climatically suitable. Areas currently climatically suitable are shown in grey, those projected to be climatically suitable by **at least** one of the six climate models by 2050 are shown in pink and by 2080 in red. The overlap of the current (grey) and the projections to 2050 or 2080 can be seen in dark red. Black indicates the areas where **all** climate models agree that currently suitable climate will persist by 2080.

All models indicate a strong contraction of climatically suitable area for lowland Themeda grassland in Tasmania by mid-century (2050) (Fig 1a). Half of the climate models show no suitable climate remaining for GTL by 2080 and the remaining three suggest very strong contractions. These areas only overlap 0.1–0.3% of the current distribution (shown in black in Fig 1).

6516 ha (87%) of GTL are currently considered to be in good condition (Table 2). All climate models agree that 99% of the good condition GTL grasslands will have unsuitable climate by 2080 (Table 3). Three models suggest that no good condition GTL will remain within suitable climate, two models show between 23 and 25ha will remain suitable, and one model projects only 8ha will remain (multi-model mean 10ha). However, these small areas that are projected to become climatically suitable in the future occur outside the current geographic range of lowland native grassland.

**Table 3:** Area of current good-condition Lowland grasslands that are projected to remain climatically suitable by each climate model by 2050 and 2080.

	Area (ha)		% of current good condition		% of all good condition grassland
	GTL	GPL	GTL	GPL	(LNGT)
**Area projected to be unsuitable by all models (2050)**	6020	3886	92	49	69
**Area projected to be suitable (2050):**					
MIROC3.2 (medres)	182	508	3	6	5
ECHAM5/MPI-OM	32	1757	1	22	12
GFLD-CM2.0	83	2920	1	37	21
GFDL-CM2.1	234	1408	4	18	11
UKMO-HadCM3	123	434	2	6	4
CSIRO Mk3.5	89	2149	1	27	16
Multi-modal mean	124	1528	2	19	12
**Area projected to be unsuitable by all models (2080)**	6465	3452	99	46	69
**Area projected to be suitable (2080):**					
MIROC3.2 (medres)	0	175	0	2	1
ECHAM5/MPI-OM	8	818	0.1	10	6
GFLD-CM2.0	0	424	0	5	3
GFDL-CM2.1	25	1351	0.4	17	10
UKMO-HadCM3	0	763	0	10	5
CSIRO Mk3.5	23	4232	0.4	53	30
Multi-modal mean	10	1294	0.2	16	9

#### Lowland Poa Grassland community (GPL)

The area climatically suitable for GPL by 2050 and 2080 is also projected to contract strongly, although a slightly larger area remains suitable compared to GTL. There are small areas within the current distribution that are projected to remain suitable for GPL by the end of the century by at least one model (Fig 1b).

7920 ha of GPL are currently considered to be in good condition (Table 2). All models agree that 46% of the current good condition GPL grasslands will no longer be climatically suitable by 2080 (Table 3). The extent of GPL projected to have suitable climate by 2080 varies between models (range 2–53%, multi-model mean 16%).

#### Natural Grassland and closely related vegetation communities (GTL+GPL+NBA)

**Fig 2:**
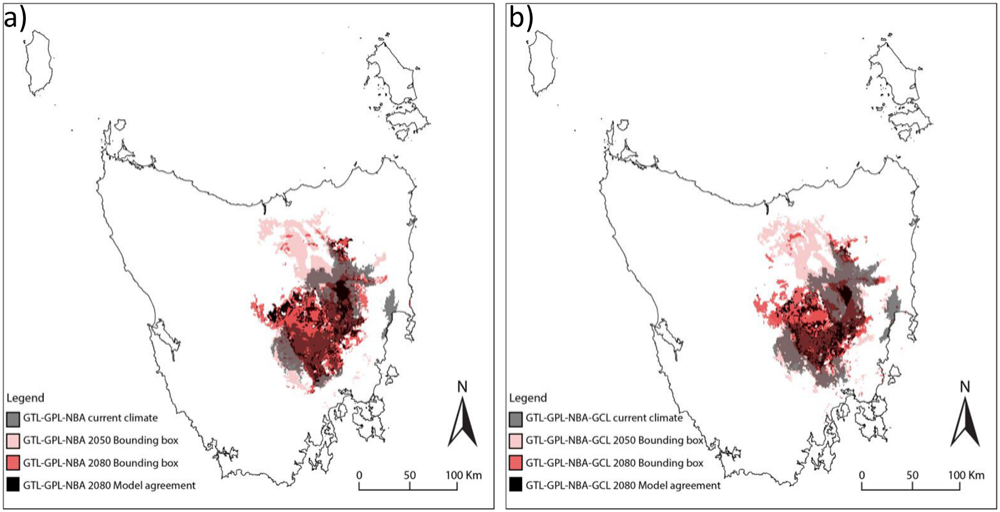
Current and future climate suitability for a) Natural grasslands and closely related vegetation communities (GTL+GPL+NBA), and b) Natural and derived grasslands and closely related vegetation communities (GTL+GPL+NBA+GCL). White represents areas that are not climatically suitable. Areas currently climatically suitable are shown in grey, those projected to be climatically suitable by **at least** one of the six climate models by 2050 are shown in pink and by 2080 in red. The overlap of the current (grey) and the projections to 2050 or 2080 can be seen in dark red. Black indicates the areas where **all** climate models agree that currently suitable climate will persist by 2080.

When we considered lowland grasslands in a broader sense by including the *Bursaria-Acacia* woodland and scrub community, we found that suitable climate contracts in the future, but some areas remain climatically suitable within the current distribution (Fig 2a).

#### Natural with derived grassland and closely related vegetation communities (GTL+GPL+NBA+GCL)

When grasslands are considered part of a single broad vegetation type that includes both natural and derived grasslands, larger areas are projected to remain suitable into the future (Fig 2b). However, as with the other grassland communities, the rate of change indicated by the difference between the grey, pink and red areas in Fig 2 is rapid, and the overlap between current and future suitable areas is very small.

#### Dominant species

**Fig 3:**
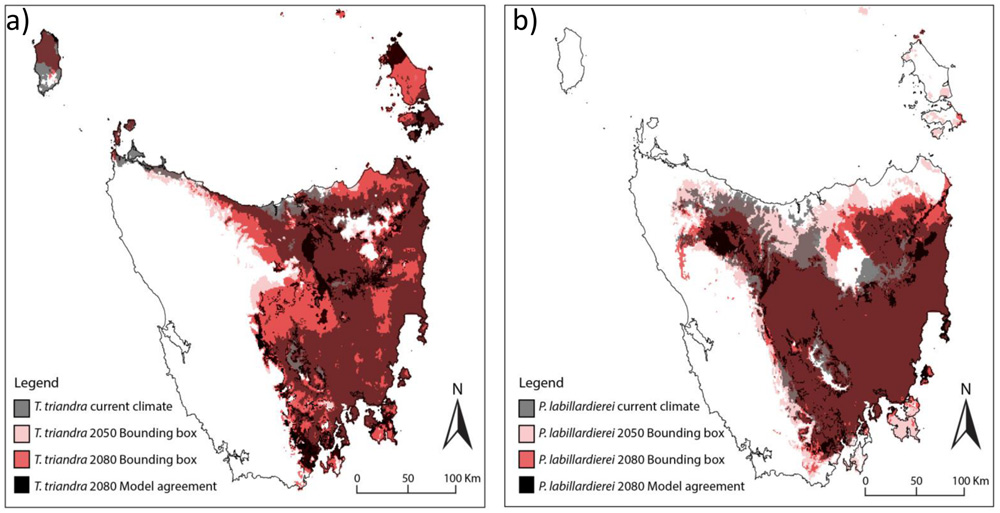
Current and future climate suitability for the dominant species, a) *Themeda triandra* and b) *Poa labillardierei var labillardierei*. White represents areas that are not climatically suitable. Areas currently climatically suitable are shown in grey, those projected to be climatically suitable by **at least** one of the six climate models by 2050 are shown in pink and by 2080 in red. The overlap of the current (grey) and the projections to 2050 or 2080 can be seen in dark red. Black indicates the areas where **all** climate models agree that currently suitable climate will persist by 2080.

The area that is climatically suitable for the grass species *Poa labillardierei* and *Themeda triandra* (Fig 3) contracts over time, but substantial areas remain suitable for 5 of the 6 climate models. One climate model, MIROC3.2 (medres), projects a very small area remaining suitable for both species. This climate model is wetter and slightly cooler than the mean of all climate models in the CMIP3 archive. The projections for *Poa* show stronger contraction than *Themeda* over the same time period, although its current extent is substantially greater.

## Discussion

Climatic suitability for lowland grasslands in Tasmania is projected to strongly contract by the end of the century, and the rate of this change is rapid. Similar changes are expected to occur in ecosystems around the world [44]. While grassland communities may persist under future climate conditions, they are likely to be in a degraded, simplified or transformed state. Conservation objectives may need to be revised to accommodate such change in management and policy development.

Attempting to maintain the status quo by conserving the current structure and composition of the grassland communities may not be a viable management option in the long term. Climatic suitability for the communities comprising the Lowland Native Grasslands of Tasmania (LNGT), the Lowland Themeda grassland (GTL) and Lowland Poa grassland communities (GPL), is projected to contract strongly by the end of the century under the A2 SRES emissions scenario considered here. Of highest concern is GTL, with almost no current grassland projected to be within climatically suitable areas by 2080. The projected future of GPL is slightly better than for GTL, but with only very small areas of good-condition grasslands expected to remain within climatically suitable areas by the end of the century. Areas that are not grassland presently may become climatically suitable in the future, but the capacity for the grassland community as a whole to establish beyond their current extent is expected to be extremely low, because native vegetation is highly fragmented within the agricultural landscape and the fine-textured soils suitable for herbaceous grassland communities are not widespread in the areas projected to be suitable [45]. Change is likely to be too rapid for many species to recolonise new areas, especially when distant from the source location. Geographic shifts to new areas will also be limited by the competitive ability of species at any future site, the influence of extreme events and by other threatening processes that affect grassland persistence.

As the climate becomes less suitable for the lowland grassland communities, it is likely that there will be a gradual change in species composition and dominance as some species are replaced by others. Species responses will differ, and occur at different times and rates. Some species may adapt to changing conditions, or cope by altering their phenology, physiology or behaviour [46–48]. Nearly 40% of the Tasmanian grassland flora is herbaceous perennials, annuals and ephemeral species that do well in open and dry environments and may have traits that convey adaptive capacity in the new conditions. Many of these component species have broad temperature and rainfall ranges (See http://www.florabank.org.au/). On the other hand, more than 10% of the vascular grassland flora is endemic to Tasmania, with highly specific habitat requirements. These species, and others with narrow thermal requirements, will be less able to tolerate temperature changes and may become locally extinct. While lags could be expected between changes in mean climate and the community response, some changes may also occur earlier in response to the increased occurrence and severity of extreme events such as droughts and wildfires [49]. We have not considered the effect of elevated atmospheric carbon dioxide (CO_2_) on the lowland grasslands, but different plant species also vary in their responsiveness to increasing CO_2_ [19, 50]. Differing plant responses suggests that competition between species may shift, changing both the structure and function of plant communities.

Our projections of suitable climate for the dominant species, *Themeda triandra* and *Poa labillardierei*, suggest that the lowland grasslands may become a simplified version of the present community, with these structural dominants persisting in some areas, while other, more specialised species may decline. Given their current broad distributions and thermal tolerances, these species might be expected to disperse across the landscape into areas that become more climatically suitable in the future, and are likely to persist in other community types such as grassy woodlands or forests. However, in areas where the climate is projected to become less suitable for the dominant species, the potential for exotic species to invade is high [10]. Invasion success is higher when communities or component species are under climatic stress, such as during prolonged drought. Several species of exotic grass (eg. Serrated Tussock Grass, *Nassella trichotoma*) are very successful invaders, and form dense, single-species stands. Once established, such monocultures exclude native species, altering the fire regime and functioning of the system [51]. Management of weeds will be particularly important in these areas.

We have focussed on the potential for changes to species composition, but changes to the structure of grasslands may also occur. Existing scrub communities may become more widespread in response to increased atmospheric carbon dioxide [52]. This phenomenon of increased woody plant abundance, known as “woody thickening”, has been well documented in recent decades [53–56]. Alternatively, grasslands may shift to other structural types such as *Callitris* or *Casuarina* forests [10]. On the other hand, vegetation with a grassland structure may establish where climate change leads to the transformation of other ecosystem types. For example, decline in canopy cover is projected for some areas of Tasmania (Williamson et al. 2014) and past tree decline events have resulted in the persistence of grassland communities where the original understorey was grassy.

The manner in which conservation management should respond to change will depend on the response of the ecological community at particular localities (Table 4). However, all of the management goals discussed here require a move away from the current paradigm that focuses on specific endpoints or reference states [57]. In areas where climate is projected to no longer support current communities, management should focus on maintaining ecosystem function, and actively managing grasslands that are currently in good condition. The higher floristic and genetic diversity of these grasslands is likely to confer resilience to change in the short term, and greater adaptive capacity in the long term [10]. Maintenance of ecosystem function could be achieved by minimizing further fragmentation, weed invasion and stress from other land uses, while accepting that compositional change is inevitable [58]. This is a “no regrets” adaptation option based on good land management actions that should be occurring to increase resilience regardless of climate change [59]. Applied burning or ecologically sensitive grazing may help to maintain, or even improve, grassland condition and maximise the chance for long-term persistence of a functioning, predominantly native, grassy ecosystem [60]. Rather than reserves that no longer protect valued natural assets being de-gazetted [61], we suggest that these locations should be prioritised as being areas of highest resilience, and managed to facilitate change.

**Table 4:** Implications of changing climate suitability for conservation management.

Projected Future	Management Response	Management Goal
**Listed Ecological Community**		
Most areas become unsuitable	Minimise weeds, pests and degrading land use	Maintain resilience and facilitate change into novel community with similar function
Small areas remain suitable	Prioritise largest remnants in best condition Minimise weeds, pests and degrading land use	Maintain healthy native grassland Minimise further fragmentation
Some new areas become suitable	Translocation or restoration; Active management to reduce shrub and tree growth	Restore degraded lands to native grassland structure to increase landscape connectivity; Conserve some target species of particular social or cultural value
**Closely related and derived grasslands**		
Large areas become unsuitable	Minimise weeds, pests and degrading land use	Maintain resilience and facilitate change into different or novel community
Some areas remain suitable	Fire and/or grazing to reduce woody thickening	Maintain extent of native grassy communities
Some new areas become suitable	Restoration	Restore degraded lands to native grassland structure to increase landscape connectivity
**Dominant species**		
Large areas remain suitable	Minimise weeds, pests and degrading land use	Maintain structure of grassland; improve resilience against invasive species
Some areas become unsuitable	Minimise weeds	Support transition to new native dominant species

As the climate changes, it may be possible to maintain areas of lowland grasslands by applying alternative management options to related communities, such as the *Bursaria-Acacia* woodland and scrub community (NBA) and the Lowland Grassland Complex (GCL). NBA is part of the natural continuum from grassland to woodlands, so the application of a regular, low-intensity fire regime to this community could promote grassland vegetation and reduce shrub cover. This may be appropriate within all grasslands if increased woody growth occurs, if a primary conservation objective is to maintain grassland vegetation structure. The derived grasslands, which are the result of clearing native grassy, dry woodland and forest communities, could be managed to improve movement across the landscape. Given the highly fragmented nature of the landscape, these areas may be essential to maintain connectivity for future movement of genes and species. However, these options may only be successful in maintaining depauperate examples of lowland grassland, and may not conserve the floristically and genetically diverse grasslands of greatest conservation significance.

In areas outside the current distribution that are projected to become climatically suitable in the future, the management response could include active translocation or restoration of grassland species, to increase landscape connectivity and conserve species of particular social or cultural value. However, the emphasis should be directed towards species or ecotypes expected to be more tolerant of new conditions [62]. In locations where the climate becomes unsuitable for the dominant species, weed control will be important to support the transition to new native dominant species.

Managing for healthy ecosystem function, rather than primarily for species composition, fits well within a risk management framework because there will always be uncertainty associated with projections of future climate and in predictions of how animals and plants will respond to a changing climate [49, 63]. Firstly there is the uncertainty associated with the range in climate models, each of which is considered to represent a plausible future [64]. The six models used here all perform well based on metrics of rainfall compared with observations in south-east Australia [35], yet they project different extents and locations of climatically suitable areas. The projections for the GTL community, for example, ranged from no suitable climate remaining, through to small areas remaining in different localities. These differences highlight the importance of considering results from a range of climate models to represent a range of plausible future climates and trajectories for a community or species.

However, making decisions on the basis of multiple distribution maps poses a challenge for conservation planning. The method used to combine the maps (e.g. taking the mean, the median, the bounding box or the minimum overlap of the different models) will influence interpretation of the results. This can have consequences where conservation effort is deployed across the landscape since both extent and location of projected suitable climate will differ between climate models. Over-confidence in a single map risks the possibility that conservation investments are applied to areas that do not support the target community or species under future climate. A precautionary approach to incorporate the uncertainty due to the range in climate models is to prioritise conservation in areas where model agreement is high, while acknowledging that all climate models project plausible futures [65]. In contrast, the bounding box, the sum of all areas projected to be suitable by all models, is likely to give an overestimate of the extent of future climate suitability, since each single model projects a substantially smaller area. Alternatively, considering the ‘best’ and ‘worst’ case scenarios may be a useful framework for decision making.

Future rates of greenhouse gas and aerosol emissions are another major source of uncertainty in projections of future climate suitability. We present results based on the high SRES emissions scenario (A2), which is a more likely scenario than lower emissions scenarios for the coming decades given current trajectories of global emissions [66]. We are currently tracking at the higher end of the A2 scenario [39] and even if drastic mitigation measures were achieved now, we will be dealing with the ‘committed warming’ that is now built in to the Earth’s climate system. However, while current emissions restrict the likelihood of the different scenarios in the short- to medium term, it does not necessarily mean that the scenario will be followed to the end of the century [67]. If emissions were mitigated the changes to the grassland communities for 2080 could be expected to be similar in nature but smaller in magnitude than those presented here for the high emissions scenario.

Finally, there is uncertainty associated with the choice and parameterisation of the ecological model used. Variable choice in species distribution models can affect the projections of current and future suitability [28, 30, 31]. Using a saturated SDM has the potential to underestimate suitable area, although Maxent has been shown to be relatively robust to correlated variables [32], and attempts to avoid over-fitting by applying a regularization penalty for each term included in the model [22, 33, 34]. The inclusion of non-climate predictors may provide more realistic distributions when knowledge of the biology is available. However, climate-only SDMs have been shown to be effective and efficient for initial assessments of climate suitability compared to models based on climate and additional predictor variables [68]. We chose a climate-only approach because our objective here was to highlight the potential for changes in the climate domain of the community to occur under future conditions. If the objective were to prioritise particular areas for management, soil, slope, aspect and other biophysical data would be needed to identify suitable habitat within the climatically suitable areas.

Limitations in the available tools for understanding potential change and our inability to predict the future need to be acknowledged when making conservation management decisions [69]. In the absence of objective tests of model accuracy [28], it is important that the limitations of SDMs in general are understood, and the influence of particular modelling decisions are acknowledged and incorporated into conservation decision making. There are many factors that are known to be important determinants of species distributions that SDMs cannot consider, including dispersal, potential adaptation, species interactions, and potential responses to elevated carbon dioxide [70]. With the cascading uncertainties inherent in predicting responses to future conditions, SDMs are just one tool among many that can inform decision making by highlighting the potential for change to occur [71].

Current approaches to conservation covenants and biodiversity offsets were not designed to cope with uncertainty, or with changing boundaries or composition of community types. Mechanisms for protecting grasslands currently include formal reservations with fixed boundaries on title. Long-term conservation covenants have also become important conservation policy instruments, whereby landowners forgo environmentally detrimental land-use rights, often in exchange for financial support, while retaining ownership of the land [72]. These reserves and covenants are legally required to be managed under a certain regime, with management success judged by indicators such as the abundance of listed species and floristic composition. Our results suggest this may no longer be appropriate under climate change. Legal and policy instruments therefore need to be more flexible to allow for changes in external factors that are not under management control, such as climate change [73]. Flexibility could potentially be enabled through supporting regulations. However, safeguards will be required to ensure that increased flexibility does not weaken the protection of species rich communities of high conservation value. Management approaches that are outcomes-based rather than prescriptive may be helpful and allow for innovation. For example, stewardship payments to farmers in return for conserving biodiversity on their farms may be more flexible, and ultimately successful, than proscriptive, regulatory approaches (for example, see the Midlands Conservation Fund (http://www.bushheritage.org.au/places-we-protect/state_tasmania/tasmanian-midlands)).

A more flexible regulatory and policy environment could facilitate the protection and restoration of grasslands by allowing their transition into transformed or even novel ecosystems. If management objectives include retention of grassy ecosystems, then flexibility about their composition will be required. For example, the current listing under Australian national environmental legislation sets thresholds not only for the degree of weediness and tree cover, but also floristic composition. By the end of the century there may be few grasslands matching the description of the ecological community listed in 2009, but there may well be healthy native grassy vegetation, perhaps with no current analogue, that could be considered a conservation priority.

Our results highlight some fundamental questions about the conservation of threatened communities over the next few decades. While we know that substantial changes are likely, we do not know what will replace these communities as their climate envelope becomes unsuitable. We do not know whether the changes will be gradual and incremental or occur as a sudden threshold shift. We do not know exactly where grasslands of high conservation significance will remain. However, we do know that changes in species composition will occur, and the distribution and composition of ecological communities will shift. This suggests that the current focus on single-species conservation and maintenance of composition should be revised. Prioritising locations of high conservation significance, rather than species or communities defined by their species composition, might better conserve ecological processes and function into the future. The concept of the ecological community will remain useful in conservation if it is seen as a dynamic, rather than a static entity, in which ecological processes and functions are sustained and contribute to patterns of diversity across the landscape.

## Conclusions

The effective conservation of threatened ecological communities into the future requires some knowledge of where climatically suitable habitat is likely to persist under changing climatic conditions. When projections suggest that there may be no suitable area for a community in the future, management objectives need to be reassessed, and alternative options developed, with the dual aims of protecting high conservation value species as components of different communities, and protecting ecosystem function.
